# Open wide: Identifying North Atlantic right whale feeding behaviour using camera-validated kinematic data and machine learning

**DOI:** 10.1371/journal.pone.0352346

**Published:** 2026-09-16

**Authors:** Jay Kirkham, Kimberley T.A. Davies, Heather J. Foley, Chris Zadra, Rhyl Frith, Paolo S. Segre, Sarah M.E. Fortune

**Affiliations:** 1 Department of Oceanography, Dalhousie University, Halifax, NS, Canada; 2 Department of Biological Sciences, University of New Brunswick, Saint John, NB, Canada; 3 Northeast Fisheries Science Center, NOAA Fisheries, Woods Hole, Massachusetts, United States of America; 4 Ocean Alliance, Gloucester, Massachusetts, United States of America; 5 Department of Natural and Applied Sciences, University of Wisconsin-Green Bay, Green Bay, Wisconsin, United States of America; James Cook University, AUSTRALIA

## Abstract

Whales are at risk from human activities and behavioural information can be important for informing risk policy. Underwater behaviour is increasingly being characterised with camera tags. Here, we characterise the kinematic signature of endangered North Atlantic right whales during feeding in high-risk areas. Customized Animal Tracking Solutions (CATS) tags were deployed (n = 23) to record the body position and movement of while simultaneously recording underwater video. Manual validation of tag camera footage was used to train classification learners, with a Support Vector Machine (SVM, 95% test accuracy) being the best performing. The SVM confirmed previously identified kinematic signatures important to identifying ram filtration. where decreased swim speed and increased fluke stroke occur due to increased drag from the opened mouth. Individual whales were predicted to spend 11–76% of their time actively filtering prey, averaging 11.4 hours day^-1^ feeding. Through video audit, we observed right whales feeding close to the seafloor during the day; our model also predicted previously unreported feeding close to the surface at night. This night feeding was underestimated by traditional feeding classification using dive shape relative to both audit (43.1%) and SVM-predicted feeding (54.9%). Based on pseudotracks, tagged whales collectively spent 74.6% of their time within a vessel exclusion zone. This research provides standardised techniques for classifying behaviour and also highlights potential gaps in policy-relevant information, such as elevated vessel strike risk at night.

## 1. Introduction

Understanding feeding behaviour is fundamental not only to the ecology of marine species [1–3] but also for identifying spatio-temporal overlap with human activities [4–8]. Like most marine megafauna, the vertical and horizontal distribution of individual whales is driven primarily by prey availability and feeding [9–11]. Low trophic level rorqual and balaenid species that feed on zooplankton and small forage fish [9,12] are vulnerable to human threats as global climate change is driving pronounced shifts in prey distributions, resulting in altered species ranges and increased variability in habitat-use patterns [13]. Whales must contend with increased overlapping human activity such as shipping, resource extraction and fishing, that is potentially exacerbated by these habitat-usage shifts [14–17].

Identifying whale feeding is not straightforward. While some species exhibit regular surface feeding or highly coordinated, discrete subsurface behaviour such as lunge-feeding fin whales [3,18] or humpback whale bubble netting [19], most feed at depth where visual confirmation of feeding is not possible [9,20]. The seasonally variable ontogenetic vertical distribution and diel vertical migration of calanoid copepod prey further complicates building an accurate picture of foraging [21,22]. Rather than relying on visual observations, animal-borne biologging tags offer a detailed record of subsurface behaviour. These tags vary in function, resolution and duration. At their simplest, a tag may record time, depth, and cartesian location using satellite telemetry or GPS that is then used to infer foraging by area-restricted search (e.g., [23]) and dive shape (e.g., [18]), which are common proxies. Many studies that use lower resolution tags seek to classify foraging broadly, encompassing not just the period of active filtration at depth but also transit to and from prey patches [2,10,12,23–25]. Inferring feeding time from a period of foraging may overestimate actual time spent ingesting prey by obscuring intermittent feeding, near-bottom exploratory phases, non-feeding subsurface activity, and may fail to identify near or at-surface feeding events, thus underestimating consumption.

High-resolution kinematic data provides the ability to identify feeding, avoiding generalisations about periods of behaviour. Such data can capture tri-axial movement at over 200 Hz [26], from this, kinematic signatures can be used to identify the moment of mouth opening and closure, allowing for refined identification of filtration time [3,9]. However, studies using such data are often restricted to small geographical areas, low sample size and short attachment times (e.g., 1–3 hrs [18]). While it is possible to identify kinematic patterns manually, this approach is labour-intensive and requires a high level of expertise and concurrent video data for validation [11,20,27]. Given the enormity of high-resolution kinematic data and the near-endless derivative metrics that may be constructed from them, the application of simple models and thresholds is unlikely to fully capture the complexity of any kinematic signature. Machine learning techniques have grown in popularity in recent years with applications to classify behaviour in cetaceans [28], pinnipeds [29], penguins [30] and elasmobranchs [31,32] providing improved opportunities for semi-automated workflows.

The North Atlantic right whale, hereafter NARW (*Eubalaena glacialis*), is listed by IUCN as critically endangered [33] and seasonally occupies coastal waters of the eastern U.S. and Canada in the western North Atlantic Ocean. Species recovery has been hampered by high rates of anthropogenic mortality, primarily caused by entanglement in fishing gear [4] and vessel strike [5]. Following climatic shifts in the mid-2010s, NARW presence in the Gulf of St. Lawrence, Canada (GSL) increased, leading to a sharp rise in human-caused mortalities and the declaration of an unusual mortality event [34,35]. As such, existing management measures based on presence/absence data can be better tailored to protect NARW while minimising impacts to industry with more information on animal behaviour.

NARWs have one of the largest predator-to-prey ratios in the animal kingdom, relying almost entirely on late-stage copepods of the genus *Calanus* [1,36,37]. NARWs are known to feed using a combination of surface (skim) [5,38,39] and subsurface feeding [1,2,40]. However, in the GSL, few observations of skim feeding have been made [41], which likely reflects the lack of a daytime surface prey layer [37]. Oceanographic studies in the area have indicated a prey maxima layer close to the seafloor during daytime, that is hypothesised to be the target of NARWs [37,42,43]. This layer is primarily composed of both active and diapausing *Calanus* spp*.,* from June through October [30].

Kinematic descriptions of presumed feeding exist for some balaenids [2,44], but none have been definitively characterised, and no reliable automatic detection tool currently exists. Unlike rorquals (e.g., blue, fin and humpback whales) that use dynamic feeding strategies like lunge-feeding [3,9], balaenids employ passive (ram) filtration [2,3,45–48], filtering water while swimming slowly forward using a combination of cross- and dead-end filtration [49]. This movement forces water parallel to the inner surface of the baleen plates, concentrating small (1–3 mm) prey particles behind the baleen plates, and reducing the overall flow within the mouth [46]. The mechanics of ram filtration create large drag forces that require morphological adaptations such as compact body-form and fused cervical vertebrae [50]. Previously, this high drag has been used as a proxy for feeding in bowhead whales (*Balaena mysticetus*) and NARW, whereby reduced swim speed and increased fluking rate were used as metrics to indicate relative drag [2,44,51]. These studies also noted brief pauses in fluke stroke, speculated to reflect prey processing (or swallowing) [2,44,51]. However, it has yet to be confirmed whether these metrics accurately identify feeding, as no visual validation exists.

In this study, we use inertial sensing tags equipped with an onboard camera to conduct a validation study of behaviour (when tag position allowed) and identify kinematic signatures associated with confirmed feeding behaviour in NARW. Since many factors affect the feasibility of collecting validation data, such as tag type, placement, duty-cycling, water visibility and light levels, for example, we built and tested several classifiers. The classifiers enable systematic identification of feeding behaviour, providing a standardised method to discern these behaviours in unlabelled, high-resolution inertial sensing data. This is particularly pertinent for species such as the NARW that face intense anthropogenic pressure and are the subject of many conservation policies. To achieve these goals, we aim to do the following: 1) assess the performance of machine learning tools at detecting feeding events, using tag camera data for validation; and 2) apply the model and video information to estimate feeding kinematics, feeding effort and diel feeding patterns of tagged whales.

## 2. Methods

### 2.1. Data collection

**Fig 1:**
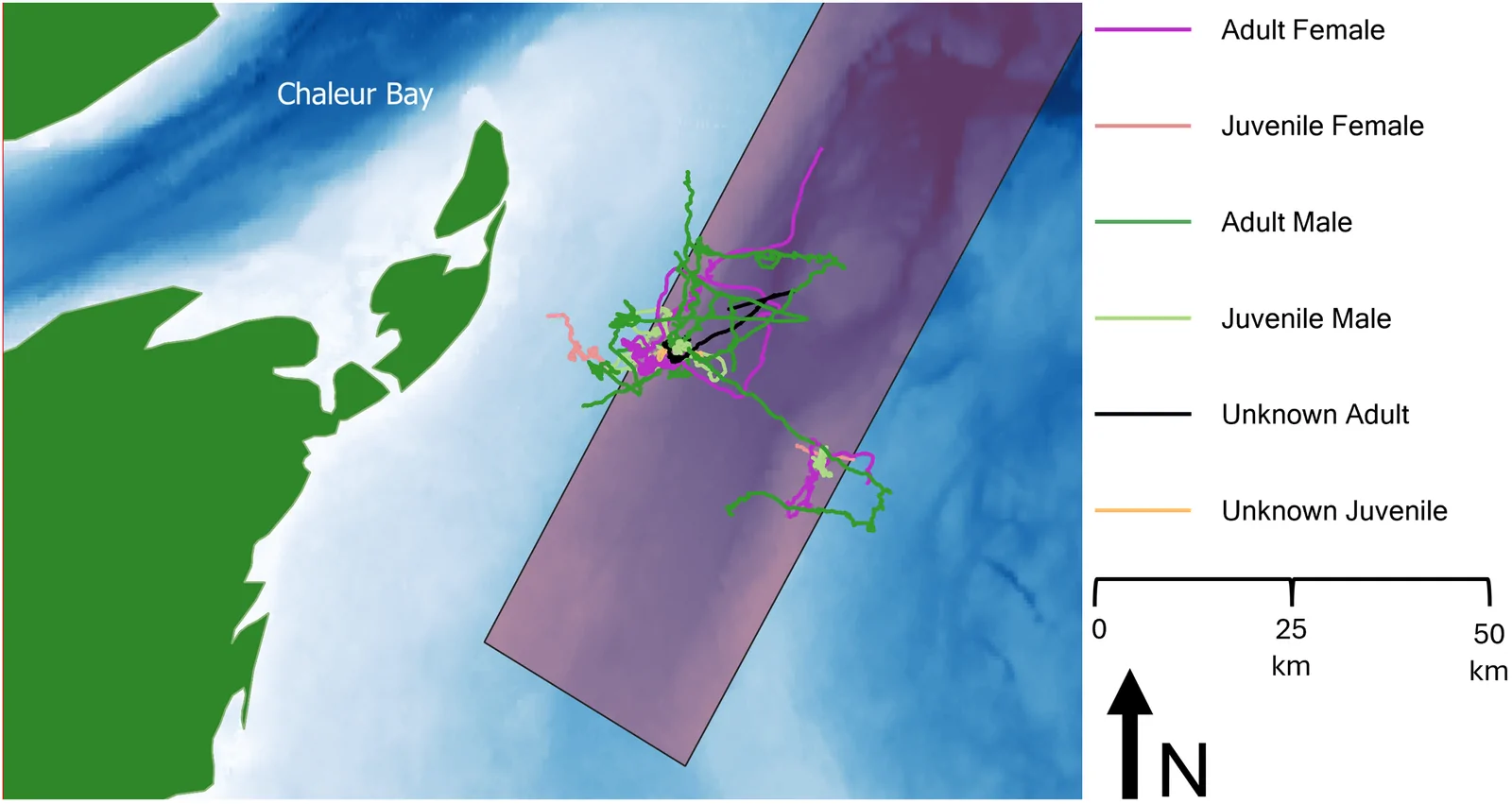
Map of tagging locations. Pseudotracks of n = 21 tagged North Atlantic right whales (*Eubalaena glacialis*) in the Shediac Valley, Gulf of St. Lawrence, Canada in July 2023 and 2024. Overlaid is the vessel exclusion zone (pink-shaded rectangle) enforced from June through September by Transport Canada. Coastline data obtained via Natural Earth (https://www.naturalearthdata.com) and bathymetric data obtained from GEBCO and interpolated [52].

Suction-cup attached inertial sensing biologging tags (Customized Animal Tracking Solutions (CATS); www.cats.is) were deployed on NARWs within the vessel exclusion zone located in the Shediac Valley, southern Gulf of St. Lawrence, Canada 14−18 July, 2023 and 5−20 July, 2024 (Fig 1 & Table 1). Tag deployments took place in water approximately 40-80m depth. Tags were equipped with tri-axial accelerometers (200 Hz sampling rate), gyroscopes (50 Hz), magnetometers (50 Hz) and depth recorders, 96 kHz HTI hydrophone and light sensors. Tags were also equipped with either CATS GPS/Satellite-linked recovery beacons (www.cats.is) or Wildlife Computers SPOT 363C (www.wildlifecomputers.com) and VHF transmitters for asset recovery. During 2023, CATS tags were deployed from a small, rigid hull inflatable using an 8 m carbon fibre pole. In 2024, tag deployment was done using a customised DJI Matrice 210 remotely piloted aircraft system (RPAS or drone) (see [53] for detailed methods). All data collection took place under DFO section 74 permits DFO-GLF-QUE-2023-01 and DFO-GLF-QUE-2024-02, with written approval from the Dalhousie University animal ethics committee. Individual whales were tagged following candidate selection criteria as outlined by Andrews et al., [54], avoiding individuals known to be mothers who recently calved, recently entangled or those in apparently poor physical condition. All tagged whales were identified using the New England Aquarium photo-ID catalogue (https://rwcatalog.neaq.org/#/). An individual identifier (EG – *Eubalaena glacialis*) number and whale name was used to identify whales, the catalogue also provides whale age, which was used for filtration rate estimation (see section 2.3.3).

**Table 1:** Individual information for tagged North Atlantic right whales (*Eubalaena glacialis*), presented with predicted individual filtration rate (volume of water filtered/ total deployment time) and proportion of time spent feeding predicted by a support vector machine model.

Dataset	Individual ID	Age (y)	Estimated Length (m)	Genetic Sex	Tag Duration (h:m)	Tagging Latitude (DD)	Tagging Longitude (DD)	Filtration Rate (m^3^/ hr)	Proportion of Time Feeding (% Deployment)
**Name – EG #**
**Training Data (2024)**	Tally- 4612	8	12.4	F	0:48	47.7635	−64.2326	1020	11.40
	5253	2	10.5	M	5:13	47.7812	−64.1841	3144	72.87
	4903	5	11.7	M	10:55	47.7874	−64.1915	4023	67.06
	Peregrine- 1628	38	13.6	NA	9:50	47.7694	−64.1978	3953	46.70
**2024**	1419	50	13.6	M	21:03	47.783	−64.1886	3248	31.97
	Manta – 1507	39	13.6	M	22:49	47.7563	−64.3038	4858	47.45
	Nimbus- 3812	16	13.3	M	7:03	47.7845	−64.1983	5403	64.77
	War-1812	36	13.6	F	32:54	47.7776	−64.2044	5271	49.23
	Warrior −3942	15	13.2	F	13:01	47.8033	−64.2197	4854	57.17
	5305	1	10.0	F	11:38	47.7552	−64.3051	1916	61.17
	5245	2	10.5	M	2:07	47.7855	−64.2083	1824	26.71
	Scrimshaw- 3333	21	13.5	M	0:29	47.7781	−64.1841	2173	29.05
	4991	5	11.7	F	14:29	47.9766	−64.1767	3945	59.96
	Sebastian – 4650	8	12.4	M	38:02	47.8078	−64.2	3477	53.69
	Salem – 3617	18	13.4	M	4:28	47.776	−64.1631	6594	72.07
	Smoke – 2605	28	13.6	F	2:12	47.7782	−64.212	5606	65.41
	Dune – 3351	21	13.5	M	13:06	47.7843	−64.1928	5993	69.39
	5257	2	10.5	M	6:22	47.7607	−64.3129	1679	39.02
**2023**	Wishbone – 4640	7	12.2	F	2:11	46.67288	−63.9952	5100	70.45
	Squilla – 3720	16	13.3	F	1:03	46.67383	−63.9592	9314	75.96
	Bocce – 3860	16	13.3	F	10:59	47.68583	−63.9668	4554	51.45
	5015	3	10.9	M	7:10	47.65883	−63.9698	5365	76.06

### 2.2. Data processing

**Fig 2:**
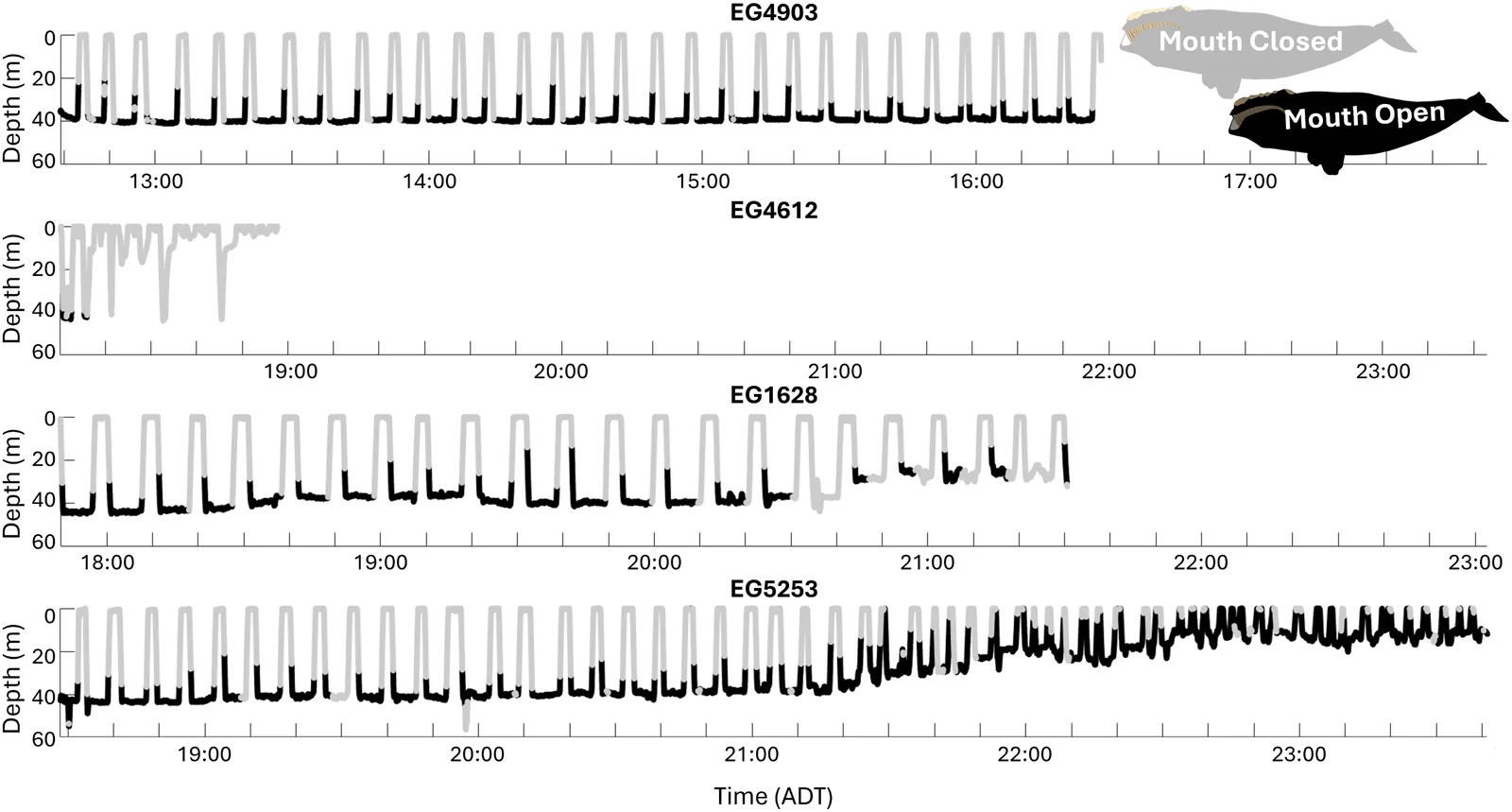
An annotated time-depth recorder for training data. Time-depth recorder data for four CATs tag deployments, manually annotated using corresponding video data to identify when the mouth was open (black) or closed (gray).

Pitch-Roll-Heading (PRH) files were created following a standardised protocol [26]. Kinematic data (accelerometer and gyroscope) were downsampled from 50–200 Hz (dependent on tag version and sensor) to 10 Hz, converted to scientific units and calibrated. Temporary detachments and reattachments, which caused orientation or position change of the tag (tag slips), were identified, and tag orientation was calibrated to match the whale’s orientation. We manually annotated feeding timestamps using a specialised behavioural analysis software, BORIS [55], as well as other notable behaviours (seafloor proximity, surfacing, conspecific interactions/socialising, body rolls and breaches) (Table 1). From the 23 total deployments, four had continuous, unobstructed views of the mouth (S1 Fig), and were used to build a ground-truthed training dataset (feeding detected 0–60 m depth). Mouth open and close times that were detectable by either a clear gap between the upper and lower jaw, or by the detection of the high contrast inner lips/orolabial sulcus, were systematically annotated for the training dataset (Fig 2). Several of the deployed tags included a light source, which allowed for video audits during darkness (either at depth or night). All analysis was conducted in MATLAB [56].

Cetacean foraging behaviour is often inferred based on dive shape, assuming right whale foraging dives have a different shape than other dive types [18,24,41,57]. A custom MATLAB script was created following the tagtools R package [58] using the CircStat toolbox [59] to summarise tagged whale dives. We used dive shape as a null model to compare our classifier to an established classification technique. This script defined individual dives and classified surface, ascent, descent and bottom phases (≥80% of max dive depth) for each dive [58]. We defined the surface as being 0–1 m depth, with dives identified when the individual crossed a 5 m depth threshold. Dive shape was then defined by the proportion of total dive duration spent in the bottom phase, whereby square dives occurred when bottom time exceeded 50% of total dive duration, U-shaped included bottom times of <50% and >20% and V-shaped dives had less than 20% of total dive duration in the bottom phase [60]. Following standard protocol for cetacean dive assessment using dive shape, we considered U- and square shaped dives as feeding [60].

### 2.3. Data analysis

#### 2.3.1. Post processing.

**Fig 3:**
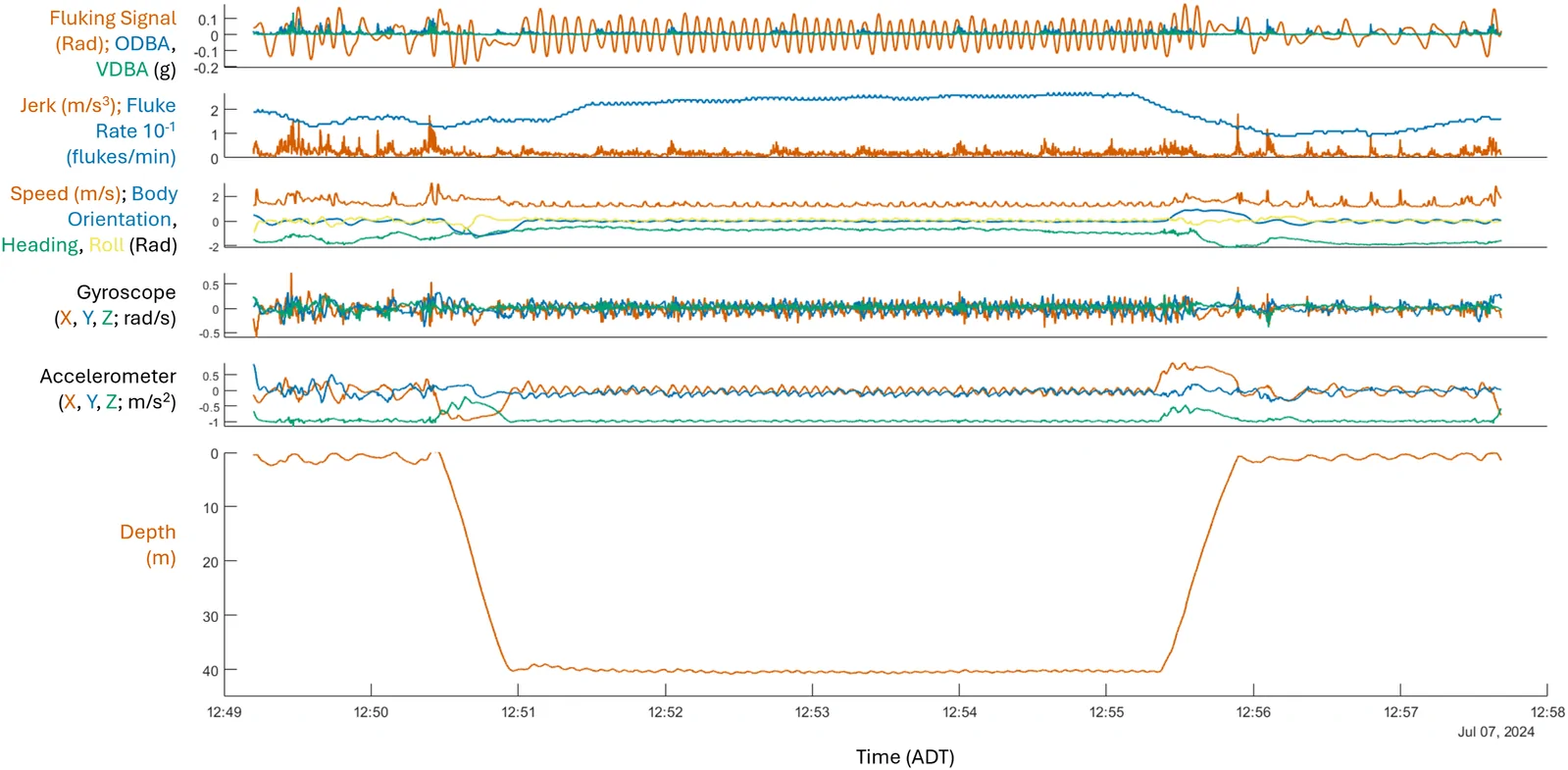
Example kinematic signals of North Atlantic right whales. Example fluking signal (radians), speed (m s^-1^) and depth over time during one U-shaped feeding dive of a North Atlantic right whale (*Eubalaena glacialis*) tagged with CATS (www.cats.is) inertial sensing tags in the Gulf of St Lawrence, Canada, from manually validated feeding event (EG4903) on 7^th^ July 2024, on EG4903. The figures show kinematics of swimming at the surface for two minutes before dive descent at 12:50:30, a ~ 4-minute dive, and after dive ascent at 12:56.

We used three sets of variables as inputs for feeding classifiers: raw, processed and post-processed variables (Fig 3). Raw variables refer to tag data that only underwent minor processing—including accelerometer and gyroscope data. These variables were then converted to scientific units and rotated to match the whale’s orientation. Processed variables included outputs from the CATs processing toolbox, including pitch (rad), roll (rad), heading (rad), speed (m s^-1^) and jerk (norm of the differential tri-axial acceleration; m s^-2^) [26]. Whereas, post-processed variables included fluking signal, fluking rate, body orientation, overall dynamic body acceleration (ODBA) and vertical body acceleration (VBA), all of which relied on the output of the CATs processing workflow [26]. These post-processed variables were calculated specifically for our modelling and are discussed in detail below.

Horizontal position of individual whales was calculated using the pseudotrack creation protocol in the CATs processing workflow [26]. This track reconstruction process uses declination angle calculated from accelerometers and magnetometers to recreate animal movement. Pseudotracks were anchored or corrected by including the location where the tag was attached to the whale and subsequently retrieved. We also included recorded locations of the tagged whale that were obtained opportunistically during the deployment. To further improve the accuracy of the track, visually observed positions from focal follows in the field, or accurate GPS or satellite locations recorded by either the CATs beacon or attached SPOT tag, are also incorporated. The tagged whale tracks were plotted and compared to GEBCO bathymetric data (www.gebco.net) and the Transport Canada vessel exclusion zone using QGIS (version 3.34.11-Prizren; [61]).

To accurately predict fluking rate, body orientation and fluking signal must be separated within the processed pitch data. Such separation allows us to understand relative swimming effort (fluking signal/fluking rate) as well as gauge body position (e.g., orienting down during descent phase of a dive) because both variables are known to be important indicators of feeding in cetaceans [2,3,44,62–64]. For this separation, we used a novel interactive plotter that allows case-by-case application of the fast-Fourier transformation (FFT) to visualise and separate fluking and body orientation [9]. A finite impulse response filter was created using user-defined input based on the FFT magnitude. The user was then prompted to highlight two main amplitudes, allowing greater inter-individual flexibility in separating fluking signal from body orientation. Previous research used static boundaries (0.2 Hz – 1 Hz; 64,65) to identify fluke stroke, which may not detect low frequency fluking as reported by Van der Hoop et al., [2] for feeding right whales. Consequently, we identify fluking outside this narrow range, and yield similar results to Van der Hoop et al., [2]. The user is prompted to highlight the fluking signal, and a bandpass filter is created to isolate the fluke stroke. The same input is used to generate body orientation via a low pass filter. The upper limit is then used as a lower limit for the fluke stroke bandpass. Fluke stroke frequency can be visually estimated based on the raw x-axis from the accelerometer or gyroscope as repeated, sinusoidal fluctuations in the axis that vary in amplitude. The periodicity of these peaks can be used to estimate the fluking rate as a manual check that calculated fluke stroke is approximately correct. Fluking rate was then calculated as a centred rolling sum of zero crossings per minute.

Whale swim speed (m s^-1^) was estimated using the amount of high-frequency tag jiggle, caused by turbulence, which increases with higher swim speeds. This was then geometrically calibrated to descent or ascent phases using pitch degree, time and depth [65]. Calibrations were made for each tag deployment following a standardised protocol that calibrates speed during dive ascents and descents using animal pitch and diving duration to calculate speed, orientation-corrected depth rate (OCDR) [65] where:


$$\begin{array}{c} OCDR=\;\Delta depth\;.\text{ sin}{\left(pitch\right)}^{-\text{1}}\;.\;\Delta {time}^{-\text{1}} \end{array}$$

To account for individual variation in swim speed capability (i.e., larger individuals likely are capable of higher speeds), as feeding predictors these estimates were used as an internally referenced relative speed. Consequently, absolute measures of swim speed were not used in our analysis. To translate raw acceleration into energy expenditure, alternatives to jerk (instantaneous tri-axial acceleration), VBA and ODBA were calculated using a Butterworth filter in MATLAB [58].

Finally, daylight classification (day, twilight or night) was estimated using solar position for the region at the given time of year. For all models, training data was split between training (70%), validation (20%), and testing (10%). The model trained on 6hr 51 min (approximately 246,600 instances of 10 Hz data), classifiers identified instantaneous feeding, whereas neural networks used 30-second chunks of data. Most training data was collected during daylight hours (76.3%), compared to night (12.5%) and twilight (11.2%) (Fig 2). Since we defined daylight to span 16 hours (66.7%), night 6 hours (25%) and twilight 2 hours (8.3%), sample sizes were biased toward daylight. This imbalance potentially makes the trained models more reliable at predicting daytime behaviour.

#### 2.3.2. Machine learning.

We trained classification learners to identify feeding behaviour from kinematic data, with 5-cross-fold validation using the interactive “Classification Learner” MATLAB app [66] to identify the most effective classifier (similar to [28]). Classifiers were iteratively given variable combinations 10 Hz depth (m), raw accelerometer (m s^-2^) and gyroscope (radians/s), processed pitch, roll and heading (radians), normalised speed (m s^-1^), jerk (m s^-3^), post-processed fluking signal and body orientation (radians), instantaneous fluking rate (flukes/minute), dynamic body acceleration (g), hour of day, and daylight classification data. No pre-determined weighting was given to the models. Additionally, we created a long-short-term-memory neural network specifically to identify feeding using the same ground-truthed dataset, following principles from human studies [67,68] (see supplementary information).

**Fig 4:**
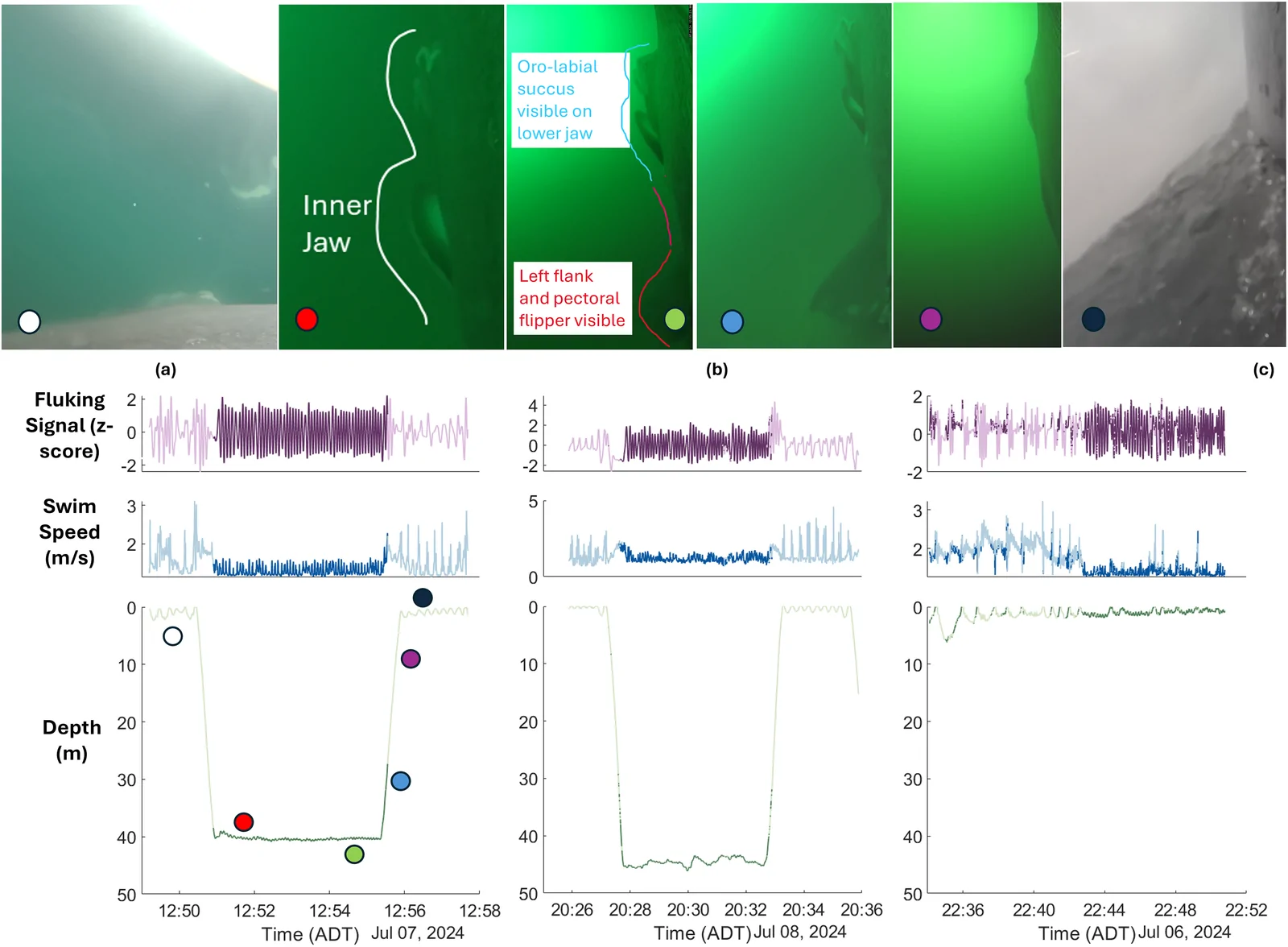
Validated and Estimated Feeding Kinematics of North Atlantic right whales- Normalised fluking signal, speed (m s^-1^) and depth (m) over time of North Atlantic right whale (*Eubalaena glacialis*) tagged with CATS (www.cats.is) inertial sensing tags in the Gulf of St Lawrence, Canada. Feeding detection (darker colour) manually annotated using corresponding video data (Panel a) or predicted using a trained Support Vector Machine (SVM). Panel (a) shows kinematic data from a manually validated feeding event on 7^th^ July 2024, on EG4903. Overlaid picture of mouth confirmed open from corresponding timestamp during bottom phase of dive, most easily identifiable by high contrast inner mouth. Panel (b) shows SVM-classified deep dive (EG2605, Smoke, 8th July 2024) and panel (c) shows SVM-classified shallow dive (EG1419, 6th July). Coloured markers on photos indicate approximate time of the screen grab from manual validation plot (panel a).


For the classification data, we normalised numeric data relative to the given deployment. Normalisation allowed us to account for individual variation differences caused by tag placement, orientation and individual size (see fluking magnitude discrepancies in Fig 4). Data were standardised using z-score normalisation where X_raw_ is a given datapoint, X̂ is the mean of that variable for the given deployment and σX is the standard deviation.

Xnorm = Xraw− X^ / σX

Principle component analysis for automated variable selection was disabled as the models tended to overfit and models were rendered invalid when applied to non-audited deployments. Following both quantitative and qualitative model selection processes (see supplementary information), we selected a support vector machine (SVM) as the best-performing model. To ensure that tag slips were not affecting feeding detection, we subset non-video audit deployments with more than three tag slips defined when generating calibrated files. We further subset these deployments by making the period between each slip a separate dataset. This additional subsetting meant that the fluking signal was determined and data were normalised per calibration period rather than using the entire deployment. We then applied our classifier to identify feeding within each subset and found that the classified feeding aligned by 94.19% with the corresponding full deployment calibration, indicating tag slips had a minimal effect on feeding identification. The nature of these slips (rotation, position on whale) was not characterised.

We assessed the effectiveness of dive shape as a classifier compared to both the audited and model predicted feeding data by calculating several metrics. We calculated false positives and false negative rates for the entire dataset and for the individual daylight classifications, whereby each row of the 10 Hz output was either a true or false (relative to audit/model prediction) positive (feeding) or negative (not feeding). An overall percentage of false negative/positive was then calculated relative to the total duration of data in the given daylight period. Gross feeding accuracy as classified by dive shape (seconds) relative to model and audit data (seconds) was also calculated for the three daylight classifications and overall, as:


$$\begin{array}{c} Gross\;Feeding=\frac{{Diveshape}_{Total\;Feeding}}{{Audit/Model}_{Total\;Feeding}}\;x\;\text{1}\text{0}\text{0} \end{array}$$

To characterise patterns in feeding by depth and daylight, we classified audit and model predicted data into discrete depth bins; deep (>20m), subsurface (5-15m) and surface (<5m) and daylight classifications. We then calculated the feeding rate (by duration) within each of these discrete bins, as well as overall.

#### 2.3.3. Filtration rate estimation.

To contextualise classifier predictions and compare our estimates to similar studies, the volume of filtered water was estimated for each tagged whale. The mouth gape of each whale was estimated based on methods from van der Hoop et al. (2019) using updated length-at-age estimates [69]. Mouth gape was calculated as:


$$\begin{array}{c} \frac{Mouth\;width\;x\;longest\;baleen\;plate\;length}{\text{2}} \end{array}$$

whereby mouth width was taken as the width at 10% of body length (m) and the longest baleen plate length (m) was estimated using data from North Pacific (*E. japonica*) and Southern (*E. australis*) right whales [2,70,71]. Mesomorphic measurements were only available for individuals up to 30 years old, consequently, for individuals older than 30 years or individuals with unconfirmed birth years (generally whales first sighted in the 1990s or earlier), age was set to 30 to ensure maximum gape size. Gape was then integrated over the distance travelled when the mouth was predicted to be open. Volume filtered was integrated throughout the tag deployment, and the proportion of time spent feeding was also calculated. To do this, the proportion of time spent feeding by each individual whale was calculated by hour of the day. We then pooled the estimated feeding time across individuals to derive an average proportion of time spent feeding per hour. We then summed these proportional hours to generate an estimate of feeding over 24 hours. This was done to remove inequalities in sampling by hour (range 4–17 deployments per hour) and account for changes in behaviour through the day. The accuracy of feeding predicted by dive shape was determined as false positive and false negative per instantaneous classification, wherein if a single point in time was classified as feeding by dive shape it would be classified as a false positive if the SVM or audit did not identify feeding. Overall, dive shape accuracy was determined on a per classification basis, whereby the total time classified as feeding was divided by the total time spent feeding as classified by the SVM or audit.

## 3. Results

### 3.1. Video audit

A video audit was conducted for all deployments, however only four had complete (or near complete) coverage of feeding behaviour (Fig 2, S1 Fig), resulting in 13hr 29 min recording time, of which 62.51% contained confirmed feeding behavior. The total proportion of deployment time spent feeding varied from 4% to 72% (Fig 2; mean + /- SD?). Feeding behaviour was observed during the day in all four training deployments, and during the night in two deployments (Fig 2 & S2 Fig). Video audits showed whales feeding near the seafloor, with the benthos visible during suspected and confirmed feeding on 50 dives (S2 & S3 Figs), with 92% of these deep dives occurring during the day. Feeding was noted during the beginning of the ascent phase of dives in most deployments (generally the first 5–15 s of the ascent phase, which equated to approximately 2–10 m of depth). This included dives without a consistent or complete view of the mouth, but where at least either a gap between lips or the inner-lip was visible as the head arched back and came into frame for tags with forward-facing cameras (S2 Fig). During behavioural analysis, feeding was also noted close to the surface during the night for one whale (EG5253; Fig 2). Another individual, EG5305, was observed diving to the seafloor (35-40m) three times at night, rolling onto its back before dragging its dorsal side along the seafloor, causing the tag to detach (S2 Fig). Other interesting behaviours were marked for all deployments and included evidence of synchronised feeding and close interactions with conspecifics (S2 Fig).

### 3.2. Classification learner

**Fig 5:**
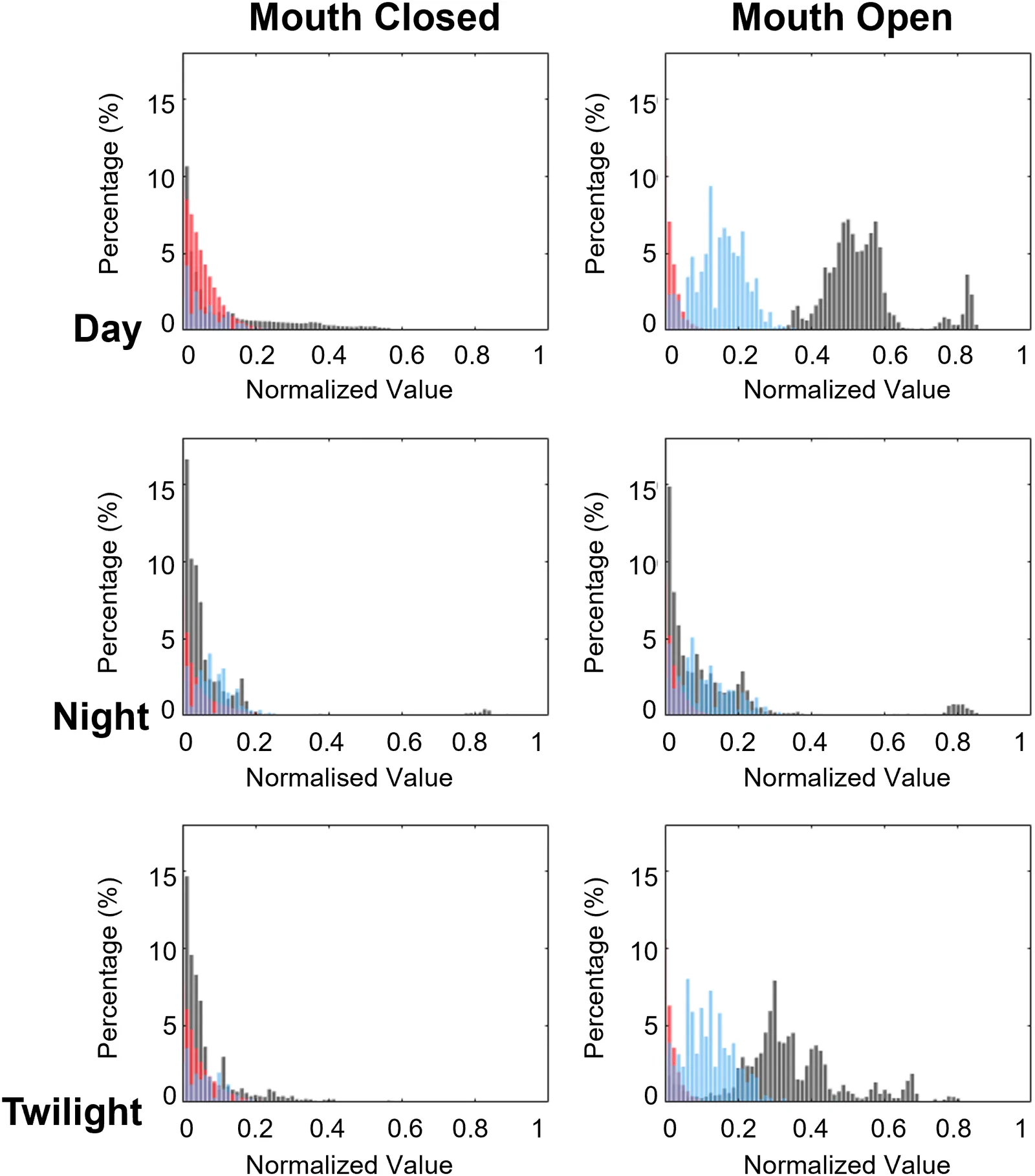
Key classification variables of feeding predicted by Supported Vector Machine (SVM). Relative density of speed (m s^-1^; red), fluking rate (flukes min^-1^; blue) and depth (m; grey) as they relate to classification of feeding by trained Supported Vector Machine model, built using audited video.

Once trained using our validated feeding dataset, our SVM predicted NARWs spent on average 11.2 ± 3.7 hours (46.6% ± 15.4 sd) per day actively feeding. The trained classifier predicted feeding mostly during the bottom phase of dives, with the mouth opening ~10 m before (during the end of the descent phase) and after (during the beginning of the ascent phase) the bottom phase of each dive for all individuals during the day. The SVM was selected to minimise false negatives, with the assumption that false positives are more likely caused by short periods when the mouth was not fully visible either due to low light or animal orientation (Table 3). We observed this feeding bracketing the bottom phase of dives during the manual behaviour audit. The SVM identified some possibly erroneous, mid-water feeding during the ascent and descent from dives. However, such behaviour was observed during non-training set video audits from deployments without a consistent view of the mouth, when the mouth was briefly visible. As expected, swim speed and fluke stroke appeared to be key kinematic features of feeding, with depth also an important predictor. The signal was strongest during the day, when whales fed at deeper depths (47.7m ± 6.0 SD) and exhibited higher fluking rates while feeding (18.7 ± 3.8 flukes min^−1^) compared to when they were not feeding (11.8 ± 5.2 flukes min^−1^) (Fig 5). At night, the differences between feeding and non-feeding depths were considerably smaller, although fluking rates remained slightly elevated during feeding (13.9 ± 4.9 flukes min^−1^) compared to non-feeding periods (11.2 ± 5.4 flukes min^−1^) (Fig 5).

Relative to both the audit and SVM-predicted feeding behaviour, dive shape identified a similar amount of time feeding during the day (98.5% relative to SVM; 100.4% relative to audit) (Table 2). However, during the night this overall feeding time was underestimated by dive shape relative to both audit (43.1%) and SVM-predicted feeding (54.9%) (Table 2). This underestimation was characterised by an increase in dive shape false negatives (73.2% relative to SVM data; 59% relative to audit data; Table 2).

**Table 2:** Predicted feeding time proportions from manually-validated and Supported Vector Machine (SVM) model output, compared to classification using dive shape [58]. We derived false positive and negative rates of feeding detection for dive shape relative to SVM and manually audited video data.

	Daylight	Dive shape False Negative	Dive shape False Positive	Dive shape/Predicted	SVM False Negative	SVM False Positive	SVM/Predicted
SVM	Day	4.2%	2.8%	98.5%	~	~	~
	Night	73.2%	51.1%	54.9%	~	~	~
	Twilight	34.1%	17.7%	80.2%	~	~	~
	Overall	18.4%	8.9%	89.6%	~	~	~
Audit	Day	6.4%	6.8%	103.8%	4.6%	17.4%	107.0%
	Night	59%	4.7%	43.1%	7.5%	42.2%	100.4%
	Twilight	22.9%	25.7%	103.8%	5.8%	43.4%	135.8%
	Overall	17%	8.7%	90.9%	5.2%	12.8%	108.7%

**Fig 6:**
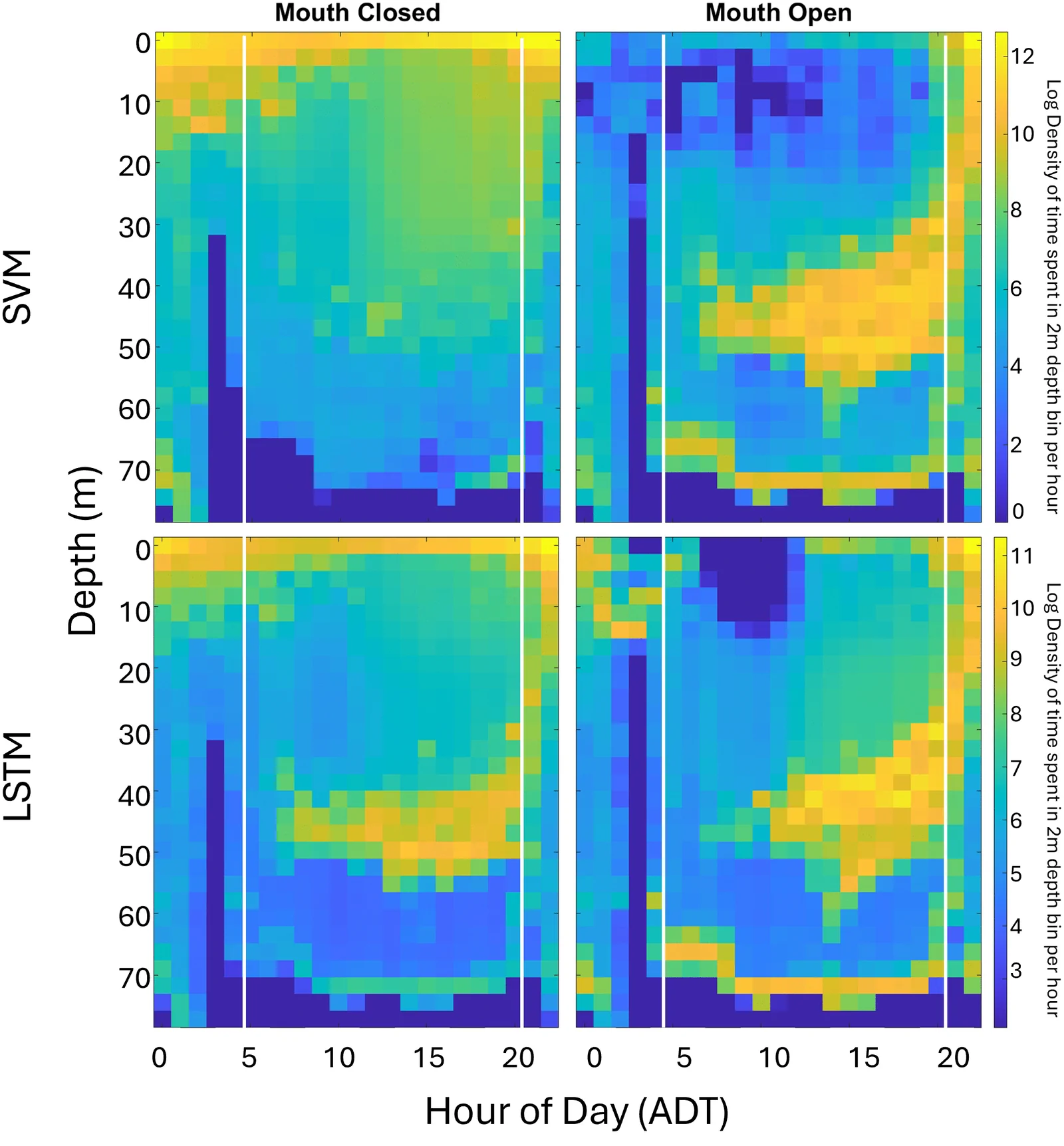
Time-depth heatmap of feeding and closed predicted by supported vector machine. Log density heatmap of North Atlantic right whale (*Eubalaena glacialis*) presence by hour of the day by 2m depth bins, normalised by total time per hour. Day-night cycles marked with white lines; twilight considered surrounding hour to sunrise/sunset. Feeding was determined through a trained Supported Vector Machine (SVM) and Long-Short-term Memory (LSTM) neural network models based on processed 10 Hz accelerometer and video audit CATs tag data.

Based on the results of the SVM, tagged NARWs spend little time with their mouths open at the surface during the day while in the Shediac Valley (GSL) in July (Table 3). Conversely, tagged whales surface feed more often early in the night (21:00–00:00) with an average feeding depth of 14.2 m ± 15.9 SD (Fig 6). This diel pattern is also reflected in the training data, for which 0.72% of daytime surface activity (<5m) was classified as feeding, compared to 54.25% of nighttime shallow activity. Most feeding was predicted to occur at depth (>20m) during the day with 93.4% of time below 20 m classified as feeding (Table 3). This prediction aligns with training data (89.2%) with confirmed feeding occurring between either 40–50 m or 70–80 m depth (Fig 6) during the day. During the night (63.2%) and twilight (90.6%), deeper dives (>20 m) were also predicted to be mostly for feeding (Table 3). Conversely, non-feeding behaviour was predicted more often at the surface throughout the 24-hour day cycle (Fig 6). Very few deep dives (>20 m) were predicted to not reflect feeding (17 out of 1442 dives) and instead may represent exploration (i.e., searching for prey) or misclassification (i.e., wrongly assigned non-feeding label).

**Table 3:** NARW feeding times predicted by Support Vector Machine (SVM) or manually audited using onboard camera. Data classified by whale depth strata and daylight classification and averaged across all available data for the given depth/daylight classification.

Model	Daylight Classification	Proportion of time feeding when at depth >20m	Proportion of time feeding when at depth 5-20m	Proportion of time spent feeding when at surface (<5m)
**SVM**	Day	93.40%	2.9%	1.2%
	Night	63.20%	46.4%	31.5%
	Twilight	90.60%	32.3%	7.2%
	Overall	92.1%	31.6%	14.3%
**Audit**	Day	89.20%	1.0%	0.7%
	Night	94.70%	95.4%	54.3%
	Twilight	74.40%	57.6%	2.4%
	Overall	87.6%	61.3%	0.7%

### 3.3. Filtration rate estimation

The average proportion of time spent actively feeding and ingesting prey per hour based on the trained SVM was 54.4% ± 17.4 SD (range 11.4−76%). Filtration rate varied from 1,020.3 to 9,313.9 m^3^ h^-1^ and was influenced by age, the sole predictor of mouth gape (Table 1 & S2Table). Overall, tagged whales spent 11−76% of their time with their mouths open. For example, EG5257 (1,678.9m^3^ h^-1^) and EG5245 (1,824.4m^3^ h^-1^) had two of the lowest volumes filtered and foraged for 39% and 26.7% of tag deployment, respectively.

## 4. Discussion

### 4.1. Summary

Right whale feeding was identified and classified using visually confirmed kinematic data. This audited data was then used to train automatic classifiers, that could be applied to deployments without verifiable mouth opening video. Compared to these classifiers, dive shape alone is a coarse predictor of behaviour, our results show that broad classifications of dives are likely to underestimate feeding, particularly at night. Assuming prey ingestion occurs only during the bottom phase of square and U-shaped dives results in feeding-time bias relative to both ground-truth and SVM-classified data. Dive shape underestimates absolute feeding time by ~10% (9.88% compared to audited data, 11.6% compared to SVM predictions; Table 2), driven primarily by very high bias at night. When using dive shape, a depth threshold is needed to identify dives to prevent dive classifications between breathes or during prolonged surface activity (generally 5 m). As such, dive shape excludes potential feeding behaviour during shallow dives (<5 m) as well as skim-feeding [72]. This misclassification is crucial when considering prey field distribution, habitat utilisation, energetic budgets, potential anthropogenic risk exposure and management consequences. Underestimation of nighttime feeding is of consequence to management and energetic modelling [1,73,74]. To assume whales feed only at the bottom of deep dives would lead to overestimation of the importance of deep (diapausing) prey layers [37]. However, in situations when night feeding makes up a substantial portion of feeding time, then diel migrating prey layers would be more significant in energy budget calculations.

While classifiers were trained with many kinematic variables, decreased swim speed and increased fluking rate showed patterns similar to those previously used to identify balaenid feeding [2,44]. These two variables are useful not only for the statistical definition of feeding but also for visual inspection of other balaenid feeding [44]. Swim speed was slower while feeding (1.28 m s^-1^ ± 0.14 SD) and faster when not feeding (1.49 m s^-1^ ± 0.35 SD) (Fig 5). Likewise, fluking rate was higher when the mouth was open during ram filtration (18.4 flukes/minute) compared to when the mouth was closed (11.8 flukes/minute) (Fig 5). These kinematic features are likely indicative of the increased drag caused by the mouth being open [2,44]. Using this validated training data, classifiers trained with the SVM were consistently the best performing.

Most tagged whales followed a diel dive pattern (Fig 6) likely following the vertical migration of their Calanoid copepod prey [1,41,75,76]. This vertical migration of non-diapausing copepods is well-documented in the region [77,78]. However, our results are the first video documentation of NARW feeding close to the seafloor (mostly during the daytime), which has been speculated to occur in the GSL [37]. This may explain previous sightings of individuals surfacing with sediment on their rostrum in other summertime foraging grounds [4], and our observation of tags retrieved with mud. Kinematic data associated with deep, nighttime dives show decelerated swim speeds (1.36 m s^-1^ (±0.24 SD)) and increased fluke strokes (13.3 flukes/minute (±6 SD)) when feeding is predicted by SVM. The bimodal distribution of feeding behaviour during the day likely reflects spatial patterns in bathymetry, based on shipboard observations (Fig 6). When tagged whale pseudotrack locations and dive depths were compared to GEBCO bathymetric data, whales were found to be within 5 m of the seafloor for 49% of their time diving (below 5 m depth).

### 4.2. North Atlantic right whale feeding estimation

Our ability to collect, process and interpret high-resolution biologging data is continuously improved through technological advancement. Although much is known about the dynamic lunge feeding of Balaenopteridae (e.g., fin, blue and humpback whales; [3,9,11,12,20,27,62]), comparatively less is known about the kinematic signature of balaenid (bowhead and right whale) feeding.

Previous research has suggested breaks in fluking during presumed foraging bouts may be related to prey processing [2]. Our video audit indicates the mouth was continuously open throughout the bottom phase of daytime feeding dives in the Gulf of St. Lawrence. Likewise, concurrent kinematic data has indicated increased fluke rate, amplitude and reduced swim speed, all likely products of increased drag. Similar behaviour has been observed in bowhead whales, with a slow continuous fluking gait rather than stroke-and-glide [44].

It has previously been suggested that the stroke-and-glide strategy may be a mechanism to increase dive time through decreased aerobic expenditure in air-breathing subsurface feeders [44,79–83]. However, the tagged whales in our study were feeding in relatively shallow water (max ~ 77m). Given previous time-depth recorder studies conducted on NARW in a deeper summertime foraging ground in the Bay of Fundy (121.2 m average depth and 12.17 min dive duration) that found whales to be diving within their aerobic dive limit [1], it is expected that they are similarly not exceeding their aerobic capacity while conducting comparatively shallower (47.7 m ± 11 SD on average during the day) and shorter duration (7.2 mins ±1.9 SD on average during the day) dives in the GSL, and consequently do not require changing their locomotion to include cost-saving stroke and glide behaviour.

During the day, when the prey layer is concentrated at deeper depths close to the seafloor, NARWs exhibited highly regular, stereotypical, near-constant feeding dives. Such dives are easily detectable via dive shape. However, at night, feeding took place in shallower water and was less discrete when viewing only TDR plots, and was almost entirely missed by feeding classification via dive shape. Furthermore, video audits showed that the mouth was opened before the bottom phase of the dive had begun (i.e., on descent), potentially pointing toward some measure of sensory prey location (Figs 2–3). The mouth was also open during the first few 10–15 seconds of the ascent on almost all deep dives (>20m bottom phase) across all deployments, as well as during the final seconds of the descent phase (Fig 2, 3). This meant that 35.4% of ascent and descent time during square and U-shaped dives was classified as feeding by the SVM, but not when using dive shape alone (1.4% total time across all deployments). Combined, these results indicate that prey ingestion may not exclusively occur during the bottom phase of a putative foraging dive, evidenced by the underestimation of feeding using dive shape alone (18.6% of all SVM feeding was missed using dive shape).

Surface and subsurface feeding exemplify the necessity for empirical classification beyond dive shape. This feeding behaviour is most often seen at night in the GSL but has been observed during the day in other NARW habitats [5,38,39]. The daytime feeding seen during July is an example of when a primitive metric, such as dive shape, may be useful to apply, given the consistent shape and periodicity of the dives. This stereotypical dive behaviour can be used to provide cautionary pseudo-validation of feeding patterns that reinforce nighttime feeding characterisation. This is in part because when defining feeding as the bottom phase of U and square-shaped dives does not identify feeding on either the ascent or descent phase, was observed during both the video audit and classified data (Fig 2). While this accounts for the error during the day, at night much of the predicted feeding occurs near the surface (<5m depth). Overall, 31.5% of surface time was identified as feeding by the SVM and 54.3% was identified during manual validation. Since characterization of dive shape requires the application of a depth threshold, skim and sub-surface feeding, observed by NARW in other habitats [5,38,39] at any time of day, will be undetected.

### 4.3. Inter-habitat filtration rates

Our filtration rate estimates were on the same order of magnitude as those previously reported for NARWs in other regions [2]. The SVM predicted proportion of time spent feeding generally agreed well with the manually classified data, the largest discrepancy we found was 7.2% from the short (47 min) deployment on EG4612. We found average filtration rates to be 4,241 m^3^ h^-1^ (± 1913 SD), 32% higher than reported by van der Hoop et al., [2], who estimated 3,211 m^3^ h^-1^ (± 874 SD). Other studies by Baumgartner et al., [1] and Kenney et al., [84] estimated NARW filter between 6,534 m^3^ h^-1^ and 6,250 m^3^ h^-1^ respectively during the bottom phase of square and U-shaped dives, which equates to 2,639.76 m^3^ h^-1^ and 4,125 m^3^ h^-1^, respectively when accounting for the proportion of total time spent actively filtering water during the tag attachment time. Savoca et al., [12] predicted NARWs filtered between 2,000 m^3^ h^-1^ and 5,333 m^3^ h^-1^ for 24-hour simulations of individual feeding rates (25^th^ percentile for a low-effort feeding day with 10 hours of feeding, and 75^th^ percentile for a high-effort feeding day, with 15 hours of feeding). These filtration estimates are based on 23 Digital Acoustic Recording Tag (DTAG) deployments on NARWs with average attachment times of 1.34 hrs (0.88–3.18 hrs), assuming all individuals were adults (13−16 m) [12]. The longer tag attachment times in our study show similar estimates for GSL with adults filtering 5,151.6 m^3^ h^-1^. Since gape was based on size-at-age curves rather than in-situ morphometric measurements, our capacity to include individual variability is limited. As a product of this, age is a major driver for filtration rates. In addition, calculating baleen filtration is likely a more complex process than simply integrating gape over distance, given the variable mouth opening height and complex hydrodynamics of filtration [49].

Once data from all deployments was averaged based on hour of the day to account for diel trends and uneven daylight sample sizes, we predicted NARWs spent 11.2 hours (46.6%) per day actively feeding. Our estimate of 11.2 hours (46.6%) of a 24h day spent feeding is closest to the lower feeding rate (10 hrs; Bay of Fundy) used by Savoca et al., [12] and is in close agreement with Baumgartner et al., [1] (40.4%) who deployed time-depth recorder tags on NARW in the Bay of Fundy and used dive shape to identify feeding. Whereas Kenney et al., [84] used all non-surface time (66%) as a proxy for feeding, which the authors suggest is likely an overestimate; they may have unintentionally accounted for nighttime surface feeding that other studies did not. (Goodyear et al., [8]) also estimated a similar proportion of feeding in the Bay of Fundy and Scotian Shelf (62.8%), who defined long dives, that were documented to occur more during the day than at night (58.2% vs 41.8%). Consequently, our classifier estimates not only match our validated data, but also previous estimates of feeding effort.

**Fig 7:**
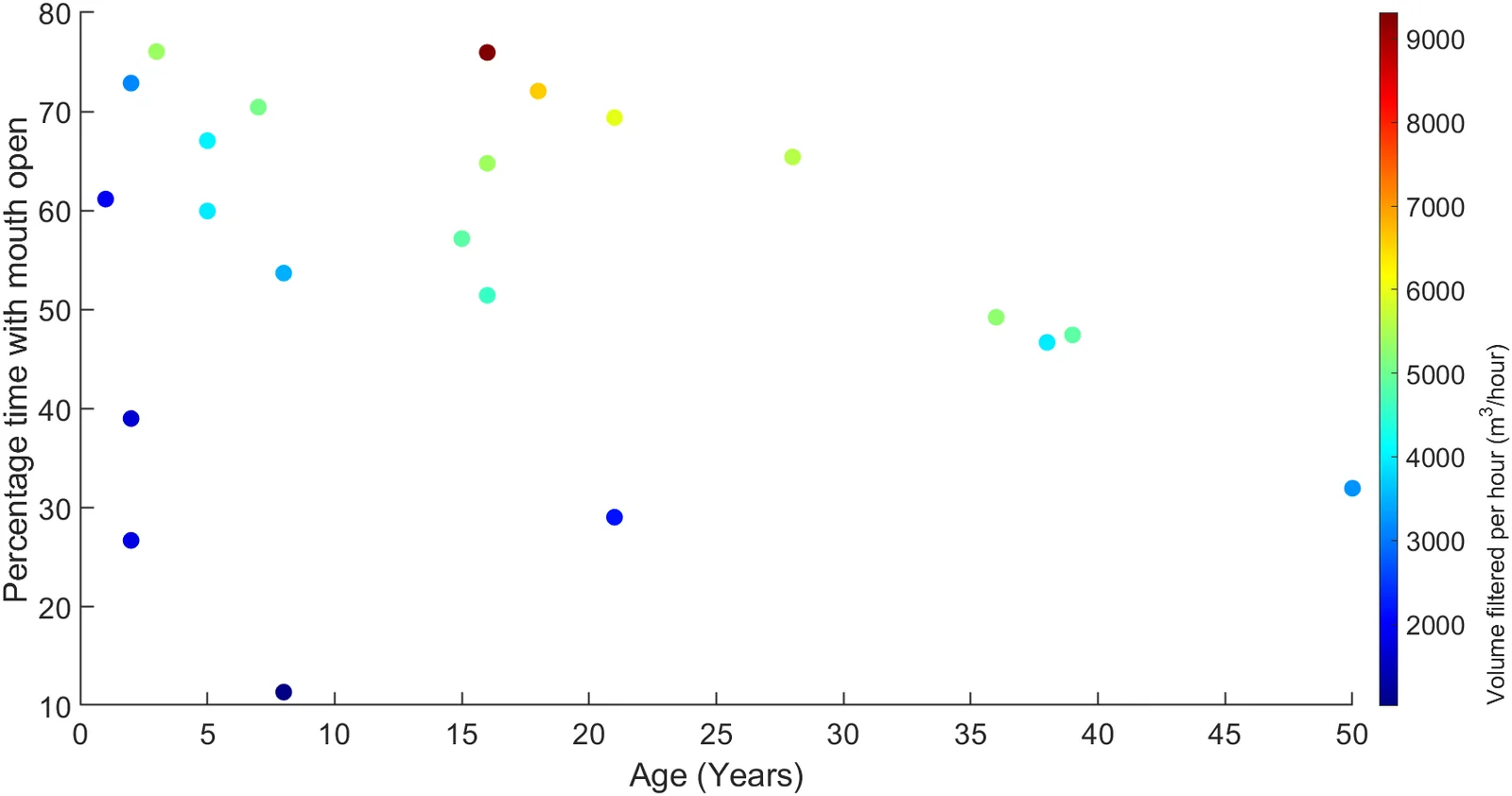
Estimated North Atlantic right whale feeding rates. Volume water filtered per hour by tagged North Atlantic right whales (*Eubalaena glacialis*) based on Support Vector Machine (SVM) trained to identify feeding, using training data from manual behaviour audit and 10 Hz kinematic data. Mouth gape was calculated per individual [2] with updated body length at age estimates from Fortune et al., [69].

Based on SVM predictions there are several outliers in volume filtered, that can mostly be explained by individual age, tag duration, and time of day. Our results show a pattern of decreased time spent feeding with age (Fig 7), which may reflect improved ability to locate prey due to experience or feeding efficiency (e.g., younger whales with smaller mouth gape). Two young whales, EG5245and EG5257, spent less than half their time feeding (26.7% and 39% respectively) and had a correspondingly small gape (S2 Table). This, along with both individuals being tagged close to sunset (20:23 & 20:04 ADT respectively), means they may have been transitioning from daytime deep feeding to nighttime shallow feeding, travelling or resting, resulting in relatively low volume filtered. Tally (EG4612; 8-year-old female), Scrimshaw (EG3333; 21-year-old male) and EG1419 (50-year-old male) also had low volume filtered likely driven by less time spent foraging. The deployments on Scrimshaw and Tally were short (29 min and 47 min, respectively) and likely not representative of a daily activity budget. The deployment on EG1419 extended throughout the night, and the SVM predicted a long period without the mouth being open that may be interpreted as rest [85]. EG3720, Squilla, spent the highest proportion of time feeding of all tagged whales, also averaging the highest speed while feeding. While these may be, in part, a product of tag placement and short deployment time (1 hr 3 min), it is interesting to note that Squilla calved two years prior to tagging (in 2021) and would likely have been building energy reserves post-weaning.

### 4.4. Management implications

The observed diel feeding patterns show that during July whales in the Gulf of St. Lawrence spend more time close to the surface during the night than during the day (Table 3 & S2 Table). If ship strikes are more likely at night, then mitigation measures, such as marine mammal observers and aerial surveys, may prove ineffective. Based on estimated whale tracks, those tagged in this study cumulatively spent 74.6% of their time within the active exclusion zone (Fig 1) and were thus less protected a quarter of the time. Whales spent a higher proportion of time within the exclusion zone during night (84.3%) than during the day (73.6%). In the southern GSL, the snow crab fishery likely represents the primary source of entanglement risk for NARWs, given its substantial spatial–temporal co-occurrence with whales and the confirmed attribution of several entanglements to this gear type [86–88]. NARW detections in the southern GSL typically begin in mid May and increase in subsequent months [86,89]. Time-area closures are triggered upon detection of a NARW, prohibiting the presence of traps with buoy lines within the region [90,91], as well as the annual cessation of the fishery on June 30 [91]. These measures are thought to reduce the risk of entanglement to NARWs by 62% [92]. Furthermore, while it has been suggested that an open mouth increases the risk for NARWs to become entangled in lines that are near the seafloor (i.e., horizontal groundlines in trawl fisheries) [4], the southern GSL snow crab fishery employs single traps with single, vertical buoy lines stretching from the trap to the surface [17,93]. These buoy lines are thought to contribute the most to entanglement risk [93,94], but are prohibited during the summer in the Shediac Valley [95,96].

### 4.5. Classifier performance

A medium Gaussian SVM was chosen as the ‘best’ model based on high test and validation metrics, as well as visual assessment, once applied to non-training data. The human validation phase identified the medium SVM as the preferred model, this human validation was important to avoid model selection that had overfit either depth, time of day, or had fit too much (mouth constantly open for hours at a time without closing) or too little (no feeding for hours, including repeated dives to similar depth, indicating prey targeting) feeding (examples for each in S3 Fig). Such overfitting in the other trial models was likely a product of the relatively small training dataset by machine learning standards [29,31,97,98].

SVM classifiers have been trained to describe behaviour from fine-scale biologging data for other marine species including teleost [99], seal, [98] and penguin [30] as well as terrestrial species [97,100]. For example, when trained on tags deployed on captive animals with visual confirmation of prey handling, an SVM was able to identify prey capture in wild little penguins (*Eudyptula minor*) [30]. SVMs have been noted to perform well when predicting binary responses (as present) and are particularly effective even with a small training dataset [29,30].

While depth is an important variable for discerning between feeding and non-feeding, such delineation becomes more difficult at night using any single variable (Fig 5). At night, tagged whales appear to have access to two different prey layers. A shallow layer forms early in the evening, likely comprising individuals undergoing diel vertical migration at night, while another deeper layer likely contains diapausing life-stages of *Calanus* spp. [22,37,42,76]. The inclusion of daylight as a predictor promoted flexibility in classification (Fig 5). However, feeding behaviour was consistently related to decreased relative speed and increased fluking frequency, regardless of depth and time (Figs 4 & 5).

### 4.6. Limitations & caveats

We were able to create a semi-automated detector of ram filtration for the first time, however, our model. Despite high energy requirements and near constant feeding while in summer foraging grounds, visual confirmation of a whale’s open mouth is not necessarily a definitive indication of active feeding. It is possible NARW intermittently (partially) open their mouth for sensory direction, thermoregulation (suggested in bowhead whales [101,102]), or hydrodynamic baleen rinsing; the latter may be the case with the aforementioned feeding behaviour on ascent from the bottom phase of feeding dives [48].

Dive shape offers a strong baseline to understand when feeding occurs under fixed conditions. During the day and early twilight, when NARWs dive to a consistent depth and feed repetitively, dive shape can accurately classify foraging behaviour (Table 2). However, during the night (Table 2), and while in other habitats where prey is not densely aggregated at depth [5,38,39], dive shape is a poor predictor of feeding.

Another limitation that may have affected the performance of the SVM relative to video audits is the difficulties associated with visual confirmation of feeding, which likely results in underestimation feeding (Table 2). Although periods of feeding (i.e., mouth open) were consistently identified through video audits, we likely underestimated total time spent feeding. Underestimates of confirmed feeding are likely the cause of 1) delays writing video files to the tags storage, creating a temporal gap between individual video files; and 2) camera angle and ambient light obscuring view. Consequently, we prioritised a model that minimised false negatives, with less penalisation on false positives during model selection. Overall, the trained SVM offers a more flexible, data-driven approach than dive shape, which remains a useful tool for feeding assessment under consistent conditions. This adaptive performance was highlighted by SVM identification of feeding during the initiation of the ascent during dives that was regularly recorded in video audits phase of ascent but excluded based on dive shape.

Obtaining accurate predictions of swim speed is particularly challenging near the water’s surface where physical disturbances create a high volume of noise in the data. The jiggle method may also be unreliable at speeds below 0.9 m s^-1^ [65]. However, the average swim speed of tagged whales in our study was 1.46 m s^-1^ (±0.3 SD). Swim speed is affected by individual whale size, and was consequently, speed was normalised and used as a relative measure, rather than an absolute value, to avoid individual size-influencing inter-deployment comparisons (i.e., we used changes in an individual’s swim speed as a predictor rather than absolute speed thresholds to predict feeding) [65].

An inherent challenge associated with measuring fluke stroke is that tag position on the body affects the amplitude of the signal. Given fluking is the primary mechanism of propulsion for balaenids, fluke stroke amplitude and rate are affected by behaviour, notably to overcome increased drag when the mouth is opened [2,44,46]. Despite following and refining procedures well established in the literature [26], there remains no standardized technique to account for tag position. For example, a tag attached close to the fluke would experience a higher amplitude of fluking than one closer to the rostrum. This means individual variability in fluke stroke signal is driven not just by behaviour but is also an artefact of tag location. To account for this potential bias, we used z-score normalised fluking signal for model training (Fig 4). This should have minimal impact on fluke stroke rate, however, which we calculated as zero crossings, and would still provide a useful feeding identifier when normalised to be relative amplitude [20,26,103]

Bathymetric data is an interpolated, coarse-scale dataset that lacks ground truth surveys, and at times yields discrepancies resulting in tagged whale dive depths exceeding the seafloor depths. This discrepancy may also be caused by errors in pseudotrack creation, possibly caused by inaccurate tag location data, such that pseudotracks were anchored to accurate tag on and retrieval locations with high-accuracy ARGOS locations derived from the onboard SPOT tag, or visual sightings during focal follows (average anchor points per tag: 8.6 ± 6.9 locations, range 1–37). Whales in shallower water (defined as <45m bathymetry) averaged deep dives (>20m dives) to 41.4 m depth; whereas whales in deeper water (defined as>70m), was 58.5m, possibly indicating a lower association with near benthic feeding in deeper water.

## 5. Conclusion and future work

Feeding behaviour influences an individual whale’s exposure to anthropogenic risk [4,104]. Understanding when and where in the water column feeding occurs is necessary to inform policy decisions for shipping and fisheries. This study provides a standardised, repeatable method for identifying feeding activity, without expert interpretation of complex kinematic signatures. We report the estimated feeding, representative of a demographically balanced (9 females, 12 males; 13 adults, 10 juveniles) subset of the population, based on long average attachment time (10.8 h). Currently, an SVM performs best at identifying the kinematic signatures associated with confirmed feeding. However, the flexibility and opportunity for transfer learning neural networks such as LSTMs offer may eventually become favoured, particularly as the training dataset is built upon. The presented model will also likely provide a useful identifier of ram feeding in other balaenid species (i.e., Bowhead, Southern & Pacific right whales), and with a similar training dataset could easily be applied to other ram-feeding species such as whales, basking sharks, or manta rays.

Estimating feeding times requires more complex delineation than simple metrics, such as dive shape. Such data and processing capability is becoming more widely available as technology continues to develop. This high-resolution data benefits from machine learning techniques as subtle changes in kinematic data must be consistently identified to accurately capture ram filtration. The use of manual behaviour validation provides a training dataset that should continue to grow and provide increased flexibility and performance in classifiers [105]. Our work capitalises on recent computing advancements to leverage more data streams at higher resolutions than previously used to estimate NARW feeding over a 24-hour cycle. Ultimately, understanding how, where, and when whales feed is pivotal in determining the level and type of anthropogenic risk to which they are exposed.

## Supporting information

S1 Fig Approximate tag placement and field of view for video audited CATS tag deployments on North Atlantic right whales (*Eubalaena glacialis*) in the gulf of St Lawrence, Canada. Overlaid on New England Aquarium photo identification composite body plan. Whale identification numbers from left to right #4903, tagged 07/07/2024; EG4612 (Tally), tagged 10/07/2024; EG1628 (Peregrine), tagged 11/07/2024; EG5253 tagged 13/07/2024.
(TIF)

S2 Fig Annotated images from CATs tag video diary deployments on North Atlantic right whales (*Eubalaena glacialis*) in the Gulf of St Lawrence, Canada. Reading left to right by row: (a) Annotated confirmed mouth open event from 10^th^ July 2024 deployment on EG4903. (b) Annotated confirmed mouth open event from 7^th^ July 2024 deployment on Tally, EG4612. (c) Unidentified conspecific feeding close to Warrior, #EG3942, on 13^th^ July 2024, who is also presumed to be feeding. (d) Subsurface interaction between Peregrine, EG1628 and another individual on 11^th^ July 2024. (e) Evidence of synchronised dive timing between Nimbus, EG3812, and another individual on 6^th^ July 2024. (f) Subsurface body contact between Nimbus, EG3812 and another individual on 6^th^ July 2024, presumably the same individual seen in the previous image. (f) & (g) Benthic interaction of EG5305, individual dove to the seafloor on three consecutive dives, rolled, and seemingly dragged its back on the seafloor.
(TIF)

S3 Fig Examples of likely misclassification of feeding by semi-automated classifiers. Semi-automated classifier outputs to predict feeding activity in North Atlantic right whales (*Eubalaena glacialis)* based on kinematic data collected from CATS (www.cats.is) tag deployments in the Gulf of Saint Lawrence, Canada. Examples shown as time depth recorder plots, colour coded by feeding (red) or not (blue) with likely erroneous classification as identified by qualitative assessment using expert judgement of likely whale behaviour in the region. Panel (a) Bagged trees model (99.43% validation accuracy) displays clear banding pattern on deployment on 15^th^ July 2024 on EG4650, Sebastian. Feeding identified 45-55m regardless of dive activity, likely an artefact of overfitting based on depth. Panel (b) Long-Short-Term-Memory neural network (79.3% validation accuracy) displays likely underfitting of feeding from deployment on 18^th^ July 2023 on EG4640, Wishbone. Feeding predicted only during ascent of dives, highly unlikely given what is known about cetacean behaviour and prey layer targeting. Panel (c) tri-layered neural network (validation accuracy 95.15%) shows more subtle, but still biologically unlikely, depth overfitting on deployment on 13^th^ July 2024 on EG1507, Manta. Period of? deep diving early in the? morning ~08:00 ADT, without feeding in bottom phase of dives, likely an artefact of the lack of training data during this time of day. Panel (d) wide neural network (validation accuracy 97.46%) exhibits overfitting of feeding on deployment from 8^th^ July 2024 on EG1812, War. This model fits almost continuous feeding throughout dive cycle for ~36 hours which is biologically unlikely, and does not match with manually-validated data.
(TIF)

S4 Fig Key classification variables of feeding predicted by long short-term memory neural network. Relative density of speed (m/s; red), fluking rate (flukes/minute; blue) and depth (m; grey) as they relate to classification of feeding by trained LSTM, built using audited video referenced to 10 Hz accelerometry data on North Atlantic right whale (*Eubalaena glacialis*) in the Shediac Valley, Gulf of St Lawrence, Canada.
(TIF)

S5 Fig Long short-term memory neural network schematic. Schematic of bi-layered long-short-term-memory neural network for classification of North Atlantic right whale (*Eubalaena glacialis*) feeding behaviour. Training data (data with manually validated feeding behaviour from camera footage) collected from 4 individuals in the Shediac Valley, Canada July 2024. Training used the ‘adam’ preset, with an initial learn rate of 0.0001; L2 regularisation of 0.0001; piecewise learn rate schedule; learn rate drop factor 0.1 on a period of 50; max epochs was set at 200 but was not reached with validation patience set to 100 (validation occurred every 5 iterations). Input data was 10 Hz kinematic data from CATS tags including; 3-axis accelerometer (m/s^2^) and gyroscope (rad/s); speed (m/s), jerk (m/s^3^), roll and heading (rad) (Cade et al., 2023); body orientation (rad), fluking signal (rad) and fluking rate (fluke/min) (calculated via FIR filtered pitch data); Overall and vertical dynamic body acceleration (g).
(TIF)

S1 Table Training and testing results from the classification toolbox of mouth open behaviour on tagged North Atlantic right whales (*Eubalaena* glacialis) based on 10 Hz accelerometry and video audit data. Evaluation metrics, model hyperparameters, and qualitative annotation to mark for clear artefacts or illogical fits. Logical refers to the number of non-audited deployments that are free of clear artefacts (max n = 19).
(DOCX)

S2 Table Extended individual information for tagged North Atlantic right whales (*Eubalaena glacialis*), presented with predicted individual filtration rate (volume of water filtered/ total deployment time) and proportion of time spent feeding predicted by a support vector machine model during full deployment period, and by daylight state; day (05:00–20:59 AST), twilight (04:00–04:59 & 21:00–21:59 AST) and night (22:00–03:59 AST). Individual estimated based on age per Fortune et al [69].
(DOCX)
