# Supplementary material for: Open wide: Identifying North Atlantic right whale feeding behaviour using camera-validated kinematic data and machine learning
Source: PLoS One. 2026 Sep 16;21(9):e0352346. doi: 10.1371/journal.pone.0352346 (PMC13580984; doi:10.1371/journal.pone.0352346)
Supplement: Supplementary file 1 [file pone.0352346.s006.docx]

| Model Name | Hyperparameters | Logical | | Qualitative Notes | Precision | Recall | F1  score | Validation | Test | Prediction Speed (Obs/Sec) | Training Time (Sec) |
| --- | --- | --- | --- | --- | --- | --- | --- | --- | --- | --- | --- |
| Bagged Trees | Learner type: Decision tree; Maximum number of splits: 339912; Number of learners: 30 | 13 | Lots of banding, overfitting and excessive mouth open high into the water column. | | 100.0% | 100.0% | 100.0% | 99.4% | 99.5% | 3181 | 1479 |
| Wide Neural Network | Number of fully connected layers: 1; First layer size: 100; Activation: ReLU; Iteration limit: 1000; Regularization strength (Lambda): 0; Standardize data: Yes | 16 | Generally, seems to fit too much mouth open | | 98.0% | 97.9% | 98.0% | 97.5% | 97.7% | 11909 | 13106 |
| Fine Gaussian SVM | Kernel function: Gaussian; Kernel scale: 1.1; Box constraint level: 1; Multiclass method: One-vs-One; Standardize data: Yes | 10 | Takes a long time to fit results; lots of good fits but generally underfitting | | 99.3% | 99.0% | 99.1% | 96.7% | 97.0% | 105 | 65804 |
| Medium Neural Network | Number of fully connected layers: 1; First layer size: 25; Activation: ReLU; Iteration limit: 1000; Regularization strength (Lambda): 0; Standardize data: Yes | 18 | Looks logical, some midwater feeding and night deep dives not classified as mouth open | | 95.8% | 95.4% | 95.6% | 95.7% | 95.7% | 19388 | 4197 |
| Bilayered Neural Network | Number of fully connected layers: 2; First layer size: 10; Second layer size: 10; Activation: ReLU; Iteration limit: 1000; Regularization strength (Lambda): 0; Standardize data: Yes | 17 | Lots of high mid water mouth open, seems to have issues with nighttime feeding. Diagonal banding on night deep dives. | | 95.6% | 95.3% | 95.5% | 95.2% | 95.6% | 15932 | 5633 |
| Trilayered Neural Network | Number of fully connected layers: 3; First layer size: 10; Second layer size: 10; Third layer size: 10; Activation: ReLU; Iteration limit: 1000; Regularization strength (Lambda): 0; Standardize data: Yes | 16 | Deep dives without feeding and some minor banding, mid water mouth opens and full water column feeding | | 95.2% | 94.9% | 95.0% | 95.2% | 95.2% | 9702 | 8144 |
| Medium SVM | Kernel function: Gaussian; Kernel scale: 4.4; Box constraint level: 1; Multiclass method: One-vs-One; Standardize data: Yes | 18 | Takes a long time to fit results; mostly logical, some feeding high into water column | | 95.6% | 94.6% | 95.1% | 95.0% | 95.0% | 71 | 36971 |
