# Supplementary material for: Open wide: Identifying North Atlantic right whale feeding behaviour using camera-validated kinematic data and machine learning
Source: PLoS One. 2026 Sep 16;21(9):e0352346. doi: 10.1371/journal.pone.0352346 (PMC13580984; doi:10.1371/journal.pone.0352346)
Supplement: Supplementary file 2 [file pone.0352346.s007.docx]

|  | Name - EG # | Age (y) | Estimated Length (m) | Estimated Mass (kg) | Gape Area (m^2^) | Genetic Sex | Tag Duration (h:m) | Day | | | | Twilight | | | | Night | | | | Overall | | | |
| --- | --- | --- | --- | --- | --- | --- | --- | --- | --- | --- | --- | --- | --- | --- | --- | --- | --- | --- | --- | --- | --- | --- | --- |
|  |  |  |  |  |  |  |  | **Feeding Swim Speed (m/s)** | **Filtration Rate (m3/hr)** | **Feeding Depth (m)** | **% Deployment Feeding** | **Feeding Swim Speed (m/s ± SD)** | **Filtration Rate (m3/hr)** | **Feeding Depth (m)** | **% Deployment Feeding** | **Feeding Swim Speed (m/s)** | **Filtration Rate (m3/hr)** | **Feeding Depth (m)** | **% Deployment Feeding** | **Feeding Swim Speed (m/s)** | **Filtration Rate (m3/hr)** | **Feeding Depth (m)** | **% Deployment Feeding** |
| Training | Tally- 4612 | 8 | 12.4 | 26,959 | 1.41 | F | 0:48 | 1.54 | 889 | 27.0 | 11.4% | NA | NA | NA | NA | NA | NA | NA | NA | 1.54 | 889 | 27.0 | 11.4% |
|  | 5253 | 2 | 10.5 | 16,026 | 0.94 | M | 5:13 | 1.37 | 3,089 | 40.7 | 66.8% | 1.31 | 3,030 | 29.6 | 68.6% | 1.27 | 3,634 | 13.1 | 84.6% | 1.32 | 3,265 | 28.3 | 72.9% |
|  | 4903 | 5 | 11.7 | 22,244 | 1.24 | M | 10:55 | 1.45 | 4,537 | 36.8 | 70.1% | 1.49 | 3,290 | 24.3 | 49.5% | 1.42 | 3,932 | 2.1 | 62.0% | 1.45 | 4,338 | 31.4 | 67.1% |
|  | Peregrine- 1628 | 38 | 13.6 | 35,635 | 1.94 | NA | 9:50 | 1.32 | 6,192 | 37.5 | 67.4% | 1.31 | 6,453 | 24.0 | 70.3% | 1.26 | 2,730 | 16.2 | 31.0% | 1.28 | 4,191 | 27.3 | 46.7% |
| 2024 | 1419 | 50 | 13.6 | 35,635 | 1.94 | M | 21:03 | 1.48 | 3,506 | 42.5 | 34.0% | 1.46 | 3,405 | 23.7 | 33.4% | 1.47 | 2,773 | 2.1 | 27.0% | 1.47 | 3,287 | 30.9 | 32.0% |
|  | Manta - 1507 | 39 | 13.6 | 35,635 | 1.94 | M | 22:49 | 1.55 | 6,184 | 44.8 | 57.2% | 1.44 | 3,484 | 27.7 | 34.6% | 1.44 | 2,791 | 7.8 | 27.7% | 1.51 | 5,008 | 38.0 | 47.5% |
|  | Nimbus- 3812 | 16 | 13.3 | 33,274 | 1.68 | M | 7:03 | 1.48 | 6,112 | 41.4 | 68.2% | 1.61 | 4,204 | 24.4 | 43.1% | 1.54 | 6,413 | 2.1 | 69.0% | 1.51 | 5,913 | 32.9 | 64.8% |
|  | War-1812 | 36 | 13.6 | 35,635 | 1.94 | F | 32:54 | 1.59 | 6,543 | 47.7 | 58.8% | 1.53 | 2,295 | 28.1 | 21.5% | 1.51 | 3,365 | 7.7 | 32.0% | 1.57 | 5,392 | 41.0 | 49.2% |
|  | Warrior -3942 | 15 | 13.2 | 32,829 | 1.66 | F | 13:01 | 1.5 | 6,064 | 42.8 | 67.8% | 1.59 | 2,102 | 32.8 | 22.1% | 1.58 | 3,845 | 8.5 | 40.6% | 1.53 | 5,216 | 36.1 | 57.2% |
|  | 5305 | 1 | 10 | 13,737 | 0.74 | F | 11:38 | 1.22 | 2,255 | 38.2 | 69.4% | 1.16 | 1,361 | 22.8 | 43.9% | 1.23 | 1,495 | 5.5 | 45.5% | 1.22 | 1,986 | 31.1 | 61.2% |
|  | 5245 | 2 | 10.5 | 16,026 | 0.94 | M | 2:07 | 2.03 | 954 | 10.8 | 13.9% | 1.81 | 1,901 | 4.0 | 31.0% | 2.02 | 2,327 | 1.1 | 34.0% | 1.93 | 1,741 | 4.2 | 26.7% |
|  | Scrimshaw- 3333 | 21 | 13.5 | 34,709 | 1.79 | M | 0:29 | 1.2 | 2,292 | 6.1 | 29.7% | 1.18 | 2,017 | 2.7 | 26.6% | NA | NA | NA | NA | 1.19 | 2,234 | 5.5 | 29.1% |
|  | 4991 | 5 | 11.7 | 22,244 | 1.24 | F | 14:29 | 1.55 | 4,779 | 47.7 | 69.1% | 1.53 | 4,751 | 27.3 | 69.4% | 1.49 | 2,428 | 7.8 | 36.5% | 1.53 | 4,100 | 39.2 | 60.0% |
|  | Sebastian - 4650 | 8 | 12.4 | 26,959 | 1.41 | M | 38:02 | 1.38 | 4,491 | 61.0 | 64.2% | 1.31 | 2,442 | 52.7 | 36.7% | 1.27 | 1,972 | 29.7 | 30.7% | 1.34 | 3,664 | 56.1 | 53.7% |
|  | Salem - 3617 | 18 | 13.4 | 33,983 | 1.73 | M | 4:28 | 1.52 | 6,840 | 47.4 | 72.1% | NA | NA | NA | NA | NA | NA | NA | NA | 1.52 | 6,840 | 47.4 | 72.1% |
|  | Smoke - 2605 | 28 | 13.6 | 33,523 | 1.91 | F | 2:12 | 1.37 | 6,091 | 44.5 | 64.7% | 1.24 | 5,656 | 31.8 | 66.5% | NA | NA | NA | NA | 1.32 | 5,932 | 39.7 | 65.4% |
|  | Dune - 3351 | 21 | 13.5 | 34,709 | 1.79 | M | 13:06 | 1.47 | 6,879 | 49.2 | 72.5% | 1.43 | 6,155 | 32.9 | 66.7% | 1.46 | 5,651 | 9.3 | 60.1% | 1.47 | 6,557 | 40.5 | 69.4% |
|  | 5257 | 2 | 10.5 | 16,026 | 0.94 | M | 6:22 | 1.52 | 3,460 | 33.5 | 67.3% | 1.34 | 2,204 | 24.9 | 48.7% | 1.18 | 1,238 | 4.6 | 31.0% | 1.25 | 1,657 | 15.8 | 39.0% |
| 2023 | Wishbone - 4640 | 7 | 12.2 | 25,564 | 1.36 | F | 2:11 | 1.62 | 5,580 | 55.6 | 70.4% | NA | NA | NA | NA | NA | NA | NA | NA | 1.62 | 5,580 | 55.6 | 70.4% |
|  | Squilla - 3720 | 16 | 13.3 | 33,274 | 1.68 | F | 1:03 | 2.04 | 9,382 | 52.1 | 76.0% | NA | NA | NA | NA | NA | NA | NA | NA | 2.04 | 9,382 | 52.1 | 76.0% |
|  | Bocce - 3860 | 16 | 13.3 | 33,274 | 1.68 | F | 10:59 | 1.53 | 4,673 | 49.7 | 50.6% | 1.38 | 2,053 | 4.0 | 24.6% | 1.38 | 5,815 | 2.3 | 69.5% | 1.49 | 4,632 | 36.9 | 51.5% |
|  | 5015 | 3 | 10.9 | 18,236 | 1.07 | M | 7:10 | 1.85 | 5,430 | 50.8 | 76.3% | 1.8 | 4,820 | 50.2 | 69.5% | NA | NA | NA | NA | 1.84 | 5,403 | 50.8 | 76.1% |
